# The Effect of Different Freezing Rates and Long-Term Storage Temperatures on the Stability of Sliced Peaches

**DOI:** 10.1155/2020/9178583

**Published:** 2020-11-18

**Authors:** Paul Dawson, Wesam Al-Jeddawi, James Rieck

**Affiliations:** ^1^Department of Food, Nutrition and Packaging Sciences, Clemson University, USA; ^2^Department of Mathematical Sciences, Clemson University, Clemson, South Carolina 29634, USA

## Abstract

The purpose of this research was to determine if freezing rates and holding temperatures influence peach quality during short- and long-term frozen storage. Fresh peaches (*Prunus persica*) were purchased locally, sliced, dipped in 2% ascorbic acid then drained, and packaged. The study was divided into two experiments, one to determine the effect of the freezing rate on peach quality and the second to determine the effect of frozen holding temperatures on peach quality. For the freezing rate experiment, freshly packaged peaches were placed in freezers at different temperatures (-7°C, -12°C, -18°C, -29°C, and -77°C) then removed for testing when the core temperature of the peaches reached the temperature of all freezer temperatures. The second experiment determined the long-term holding effect on quality using both fresh and prefrozen peaches held at -7°C, -12°C, -18°C, -29°C, and -77°C through 360 days. Quality measurements included freeze thaw and weight loss, lightness, firmness, moisture content, ascorbic acid equivalent antioxidant capacity (AAEAC), hexanal detection using gas chromatography (GC), scanning electron microscopy (SEM), and sensory evaluation. During the freezing phase (experiment 1), peach weight loss and surface ice crystal pore size significantly decreased with increased freezing rates. Peaches held at -77°C and -29°C maintained overall quality to a greater degree than the higher holding temperatures. However, all samples enzymatically browned when thawed; therefore, frozen peaches may best if used in the frozen state or in applications where appearance is a critical factor. In general, fresh and prefrozen peaches were not acceptable by the sensory panelists after 270 days of frozen storage.

## 1. Introduction

Since fruits grow only in certain parts of the world under certain temperature and humidity conditions and seasonally, preservation is a key factor to avail these foods to humans out of season and in regions where these fruits do not grow well. Fruits contain approximately 90% water which begin to undergo higher rates of respiration once they are harvested, resulting in moisture loss, quality deterioration, and potential microbial spoilage [1]. Refrigeration slows down the respiration of fruits to increase shelf life [1]; however, fruits continue to degrade during refrigeration. Freezing offers a retention of nutrients and other quality attributes during long-term storage. Prior to freezing, peaches are pitted, peeled, and sliced. While freezing minimizes loss of nutrients and slows enzymatic browning, browning still occurs during freezing and thawing [2]. Freezing has been successfully employed for the long-term preservation of many foods by lowering temperature to -18°C or below [3]. In fact, 6 to 8% of peaches produced are processed as frozen peaches [4]. While the holding temperature of frozen fruit is critical to retain quality, freezing rate can have an even more dramatic effect on fruit quality. Rapid freezing can prevent the loss of water from the plant cell through osmosis while slower freezing rates allow adequate time for water to migrate from the cell resulting in greater drip loss for slowly frozen fruits [2]. Because of the wall, damage during freezing, water does not return to the cells upon thawing but instead becomes drip loss [2]. Freezing fruits retard the physical, chemical, and biochemical reactions which induce phytochemical deterioration. Most of the liquid water is transformed into ice during freezing, which slows microbial, enzymatic, and lipid oxidation reactions [5]. Enzymatic reactions are a major concern in the deterioration of frozen fruits. Enzyme activity has been noted in foods stored at temperatures as low as -73°C [6]. Color loses in frozen vegetables containing chlorophyll due to pheophytisation occurs when the magnesium found in the center of chlorophyll's porphyrin ring is lost and replaced with hydrogen [7]. This reaction is caused when pH decreases during frozen storage thus initiating the pheophytisation reaction [7]. Texture is another key quality factor in fresh fruit because it determines acceptability [8]. Slow freezing rates result in large ice crystals that cause fruit cell wall damage leading to an undesirable mushy texture in frozen fruits. After thawing, fruit will be softer due to cell damage and water migration from the fruit cell structure. Phytochemicals in high concentration in fruits include phenolic compounds such as flavonoids anthocyanins, flavanols, and phenolic acids [9]. Flavonoids are primarily found in the skin of fruit, which contribute to important quality aspects such as aroma and color [10]. Since phenolic compounds are antioxidants, they are subject to oxidation during storage and processing of foods [11]. Cold storage can help retain nutritional components, for example, plums held at 0°C and had lower rates of vitamin C degradation compared with those kept at 5°C and 12°C [12]. While extensive research has shown lower temperatures slow the degradation of nutrients and other chemical degradative reactions, the difference in the rate of degradation between temperatures used in commercial freezers may not be evident. More energy-efficient freezers may be possible if research indicates that quality loss is not significant when frozen fruit is stored at slightly higher freezer temperatures.

## 2. Materials and Methods

### 2.1. Sample Preparation

Peaches (*Prunus persica*) purchased locally were rinsed with tap water, dried with a paper towel, and then cut into 8 slices. The weight of each slice was approximately 12 g to 14 g. The slices were dipped in 2% of L-ascorbic acid (Food Grade, Sigma Aldrich, Saint Louis, MO 63103, USA) for 2 minutes. The slices were then drained and packaged in Whirl Pak bags (Nasco, Sigma Aldrich, St. Louis, MO 63103, USA) then stored at 3.3 ± 2°C for 2 hours. For the long-term storage study, prefrozen peach slices were obtained from a commercial supplier and held at -20°C until use. Both prefrozen and fresh peaches were used in the long-term storage study. The size and weight of each frozen slice were approximately the same for prefrozen samples as the fresh peach slices (12 g to 14 g).

**Figure 1:**
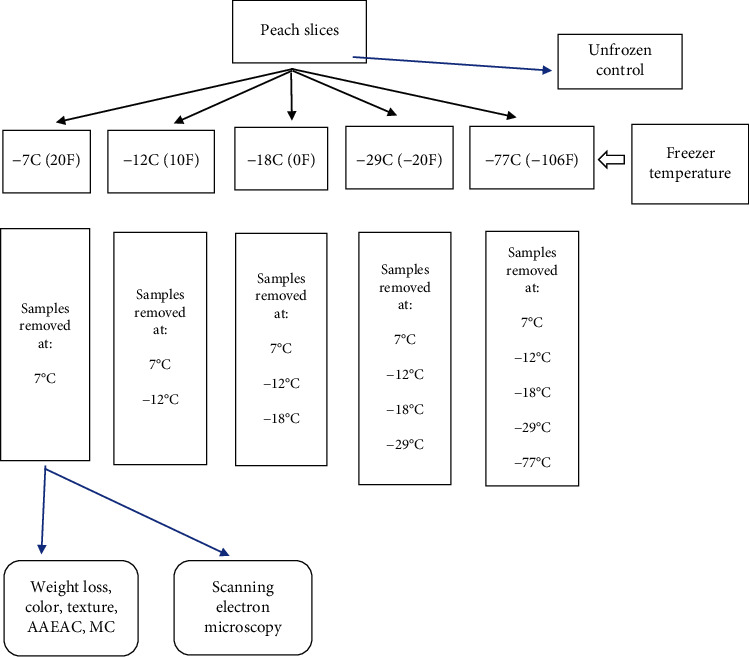
Flowchart for experiment 1 for short-term frozen storage effects on peach slice quality.

**Figure 2:**
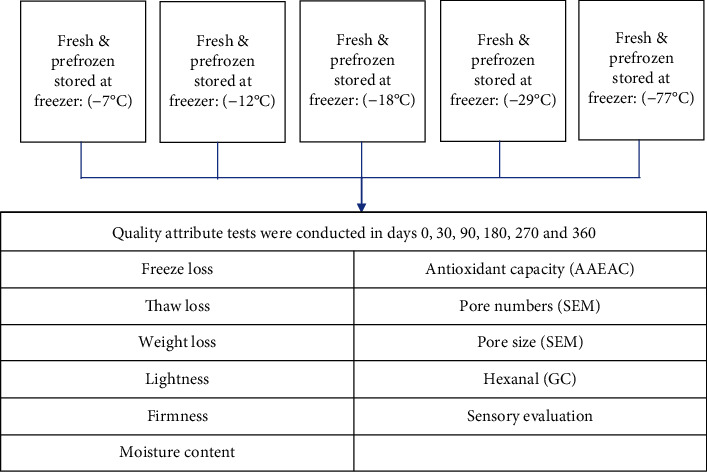
Flowchart for experiment 2 for long-term frozen storage effects on peach slice quality.

**Figure 3:**
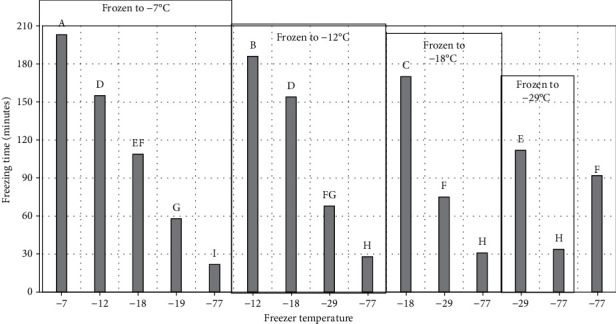
Freezing time for different freezer temperatures and endpoint core temperature. ^∗^^a-i^Treatments with different letters are significantly different (*P* ≤ 0.05). *n* = 3. Standarderror = 5.9.

For experiment 1 (short-term storage), peaches were placed into freezers having five different set temperatures (-7°C, -12°C, -18°C, -29°C, and -77°C) and then were removed for testing when core holding temperatures reached these set temperatures within each freezer. Since there were 5 endpoint temperatures for the -77°C freezer, 4 for the -29°C freezer, 3 for the -18°C freezer, 2 for the -12°C, and 1 for the -7°C freezer, this resulted in a total of 15 different treatments (Figure 1). For experiment 2 (long-term storage), fresh and prefrozen peach samples were stored randomly in freezers set at different temperatures (-7°C, -12°C, -18°C, -29°C, and -77°C) for 360 days (Figure 2). The 15 treatments resulted in different freezing times for each of the endpoint temperatures (Figure 3). Fresh peach samples were prepared as described for experiment 1 while prefrozen samples were obtained from a commercial source. The core temperature of one peach slice within each freezer was monitored throughout storage with a thermocouple, and the internal freezer temperature and humidity were recorded using a sensor. The quality attribute tests for the fresh and prefrozen peaches were conducted on days 0, 30, 90, 180, 270, and 360. On day 0, fresh and prefrozen samples were removed from each freezer set at -7°C, -12°C, -18°C, -29°C, and -77°C for testing. Two peach slices from each treatment were freeze-dried for the scanning electron microscopy (SEM) for ice crystal pore size analysis. Freeze loss and lightness (*L*^∗^) tests were measured after freezing before thawing. Other tests were conducted after thawing in the refrigerator at 3.3 ± 2°C for 24 hours.

### 2.2. Freeze Loss

Percent freeze loss was determined by weighing (Mettler Toledo PB3002 scale, Langacher Greifensee, Switzerland) fresh and prefrozen samples before each peach sample was placed into their respective freezers and then again after freezing. The percent freeze loss was calculated based on this equation:

(1) $$\begin{array}{c} \%\text{freeze}\text{loss}=\left(\text{weight}\text{before}\text{freezing}–\text{weight}\text{after}\text{freezing}\right)/\text{weight}\text{before}\text{freezing}*100. \end{array}$$

### 2.3. Thaw Loss

Percent thaw loss was determined by weighing (Mettler Toledo PB3002 scale, Langacher Greifensee, Switzerland) fresh and prefrozen samples after freezing and then again after thawing. The percent thaw loss was calculated based on this equation:

(2) $$\begin{array}{c} \%\text{thaw}\text{loss}=\left(\text{weight}\text{after}\text{freezing}–\text{weight}\text{after}\text{thawing}\right)/\text{weight}\text{after}\text{freezing}*100. \end{array}$$

### 2.4. Weight Loss

Total percent weight loss was determined based summing the percent freeze loss and percent thaw loss. The percent weight loss was calculated based on this equation:

(3) $$\begin{array}{c} \%\text{weight}\text{loss}=\left(\%\text{freeze}\text{loss}+\%\text{thaw}\text{loss}\right). \end{array}$$

### 2.5. Color

Lightness (*L*^∗^), *a*^∗^, and *b*^∗^ were measured on raw and frozen peach samples using a Minolta Colorimeter with a DP-400 data processor and CM-400 Chroma Meter (Minolta, Colorado) as described by [13]. The color analysis for frozen peach treatments was performed directly after weighing. Plastic wrap was used to cover the peach treatments during color readings to protect the Chroma Meter. The influence of the plastic wrap on color was accounted for during calibration. Color was also measured on samples after freezing and after thawing.

### 2.6. Texture (Firmness)

The firmness with and without the pericarp was determined based on the method described by [13]. A TA-XT Plus texture analyzer (Stable Micro-Systems Texture Technologies Corp, Scarsdale, New York, NY, USA) was used for the analysis with a probe diameter of 8 mm, a test depth of 5 mm, and at a penetration rate of 1 mm s^−1^.

### 2.7. Freeze-Drying Process

Two slice samples (per replication) were used for ice crystal analysis by first freeze-drying (Labconco Lyph-lock 6 Freeze Dry System, Kansas City, Missouri) to remove moisture and stabilize ice crystal pores. Freeze drying was achieved under a condenser temperature range of -44°C to -48°C and a vacuum pressure range of 50 × 10^−3^ to 500 × 10^−3^ millibars. Samples were removed from the freeze dryer after five days and stored in a 3.33°C (38°F) refrigerator overnight before microscopy analysis.

#### 2.7.1. Scanning Electron Microscopy (SEM)

The surface and core areas of the freeze-dried treatments were analyzed with a S-3400N Variable-Pressure SEM (VP-SEM) (Hitachi High-Technologies Corporation, Clarksburg, Maryland) described by [13]. The microscope was used to capture micrograph images of pores that are comparable to ice crystals formed within the tissue during freezing. Samples were first subjected to surface ice crystal damage analysis and then sliced in half to observe core ice crystal damage analysis. The instrument was set with an accelerating voltage of 20 kV and a chamber pressure of 40 Pa. Micrograph images were stored in the Quartz PCI Database of the Advanced Materials Research Lab under Clemson University.

#### 2.7.2. Image J Ice Crystal Pore Size Analysis

The Image J 1.50i software, created by Wayne Rasband and the National Institutes of Health, was used to quantify ice crystal pore damage of the micrograph images obtained from the S-3400N Variable-Pressure Scanning Electron Microscope. Each image was set to a scale of known distance of 500 *μ*m, distance in pixels of 500, and a pixel aspect ratio of 1.0. Data gathered included area, area faction, and a fit ellipse, each to a decimal place of 3. Each pore was analyzed based upon pore size from 50 *μ*m^2^ to infinity and circularity from 0 to 1.0. Based upon these settings, the software produced a total pore count, total pore area, average pore size, percent area, major and minor axis values, and pore angle data.

### 2.8. Moisture Content Determination

The peach samples were held at 100°C for 6 h, and the percentage of moisture was calculated based on the weight loss. Moisture(%) = [(weight(g)beforedrying − weight(g)afterdrying)∗100]/weight(g) before drying.

### 2.9. DPPH Free Radical Scavenging Assay

The antioxidant capacity was measured using the DPPH method as described by [14] with modifications. Briefly, fifteen grams of sliced peaches was dissolved with 100 ml of 50% ethanol and blended for 30 seconds. The samples were centrifuged at 11000 rpm/15 min at 5°C. L-Ascorbic acid standard was prepared by dissolving 0.0176 g of ascorbic acid in 100 ml of 50% filtered water and 50% ethanol. DPPH was prepared by dissolving 0.008 g of DPPH in 100 ml of 50% ethanol. 0.4 ml from the sample was put in a tube and added 2 ml of DPPH. The tubes were placed in a dark place for 30 min. 0.25 ml was taken from each tube and placed in a microplate. The absorbance was measured using EPOCH spectrophotometer at 517 nm, and the antioxidant activity was measured based on the standard curve of L-ascorbic acid concentrations. The results were calculated, and the antioxidant capacity was expressed as mg/g.

### 2.10. Sensory Evaluation

The sensory evaluation was performed as described by [15]. The evaluation was performed in individual booths by a trained panel of 8 judges. The 8 judges were trained for two days for sensory evaluation of the color, texture, and flavor compared to fresh peach control samples. Eight lengthwise slice fresh and prefrozen samples after freezing were prepared and placed onto a white porcelain dish. The samples were tested within 5 min from cutting to ensure glossiness and avoid browning reactions. Each dish containing the sample was randomly marked by different numbers. The quality attribute evaluation was based on the acceptability which was determined using a hedonic scale marked with the following two anchors: 0.0 = extremely dislike to 15.0 = extremely like.

### 2.11. Statistical Analysis

The experiments (freezing rate and long-term storage) were each replicated 3 times starting on different days using different batches of peaches. The data were analyzed using a one-way analysis of variance (ANOVA), and statistical significance was at the 5% level. For analyses where freezing had a significant treatment effect (*P* ≤ 0.05), significant differences were determined using multiple comparison tests and least significant difference (LSD) and Tukey's test for significance.

## 3. Results and Discussion

### 3.1. Experiment 1: Short-Term Freezing Effects on Peach Quality

Fresh peach slices were placed in freezers set at five different temperatures (-7°C, -12°C, -18°C, -29°C, and -77°C), then removed from each of the freezers when the core peach slice temperature reached each of the five temperatures. This created five different freezing times to reach -77°C, four different freezing times to reach -12°C, three different freezing times to reach -18°C, and two different freezing times to reach -29°C: culminating in 15 different freezing.

**Figure 4:**
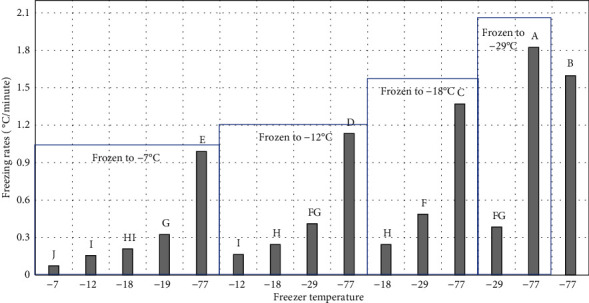
Freezing rates for treatments frozen (freezer temperature environment). ^∗^^a-j^Treatments with different letters are significantly different (*P* ≤ 0.05). *n* = 3. Standarderror = 0.03.

Freezing rate (°C/h) was calculated by the following: the difference between initial and final value of product temperature divided by freezing time [16]. This generated different freezing rates (Figure 4) ranging from 0.07°C/minute for freezing to -7°C in the -7°C freezer to 1.8°C/minute for freezing to 29°C in the -77°C freezer.

**Figure 5:**
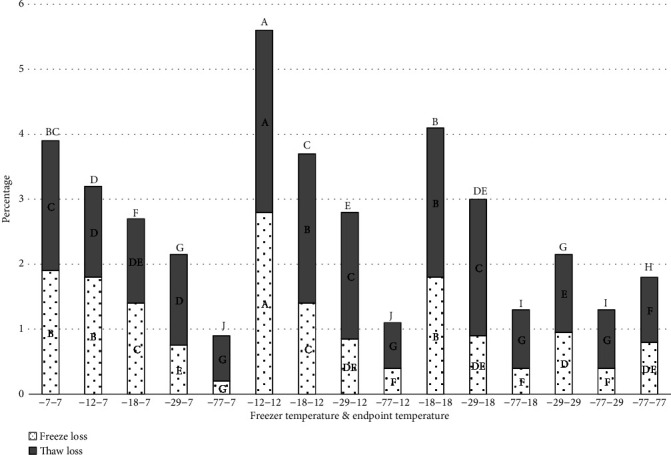
Weight loss for frozen and thawed and combined loss for fresh peaches frozen at different rates and to different endpoint temperatures. -7-7= freezer temperature is -7°C, and sample endpoint core temperature is -7°C. -12-7 = freezer temperature is -12°C, and sample endpoint core temperature is -7°C. -18-7 = freezer temperature is -18°C, and sample endpoint core temperature is -7°C. -29-7 = freezer temperature is -29°C, and sample endpoint core temperature is -7°C. -77-7 = freezer temperature is -77°C, and sample endpoint core temperature is -7°C. -12-12= freezer temperature is -12°C, and sample endpoint core temperature is -12°C. -18-12= freezer temperature is -18°C, and sample endpoint core temperature is -12°C. -29-12 = freezer temperature is -29°C, and sample endpoint core temperature is -12°C. -77-12 = freezer temperature is -77°C, and sample endpoint core temperature is -12°C. -18-18= freezer temperature is -18°C, and sample endpoint core temperature is -18°C. -29-18= freezer temperature is -29°C, and sample endpoint core temperature is -18°C. -77-18= freezer temperature is -77°C, and sample endpoint core temperature is -18°C. -29-29= freezer temperature is -29°C, and sample endpoint core temperature is -29°C. -77-29= freezer temperature is -77°C, and sample endpoint core temperature is -29°C. -77-77= freezer temperature is -77°C, and sample endpoint core temperature is -77°C. ^∗^^a-j^Treatments with different letters for freeze loss, thaw, loss and combined freeze/thaw loss are significantly different (*P* ≤ 0.05). *n* = 3. Standarderrorforfreezeloss = 0.05, thawloss = 0.06, and combinedloss = 0.09.

Peaches frozen in -7°C and -12°C freezers had significantly higher freeze and thaw loss than peaches frozen in -29°C and -77°C (Figure 5). This was expected since the freezing rate at -77°C and -29°C was significantly faster than in the -7°C and -12°C freezers (Figure 4). During the four stages of the freezing, product temperature first drops to freezing point, then before ice crystal formation begins the product temperature falls below the freezing point (supercooling) [17]. The third stage of ice crystal formation (phase change) is the longest and most energy intensive stage. The final stage of freezing is lowering the product temperature to the final storage temperature [17]. Rapid freezing leads to insufficient time for loss of water from the plant cellular structure and smaller ice crystals form within the cell compared to slower freezing rates. Slow freezing allows more water to move outside plant cell structure to form large ice crystals, damaging the plant structure resulting in greater drip loss during freezing and thawing [18]. Because of the cell wall damage during freezing, the water cannot return to the cells upon thawing but, rather, becomes drip loss [19]. Three factors that influence drip loss are high internal pressure in the product, formation of ice crystals in the product, and the irreversible removal of water from plant cells. An increase in drip loss indicates a greater loss of liquid cellular components and is exacerbated by enzyme-catalyzed disruption of cell walls and membrane damage during freezing and frozen storage [20]. Ice crystal growth may also occur during frozen storage due to freezer temperature cycling during defrosting leading to larger crystal formation, which will damage and rupture cell walls [21].

**Figure 6:**
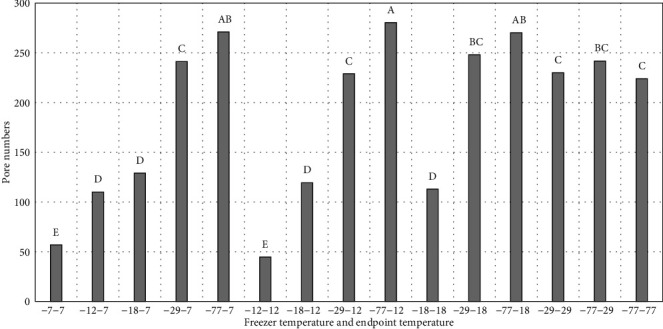
Pore numbers on fresh peach surfaces, frozen at different rates and to different endpoint temperatures. -7-7 = freezer temperature is -7°C, and sample endpoint core temperature is -7°C. -12-7 = freezer temperature is -12°C, and sample endpoint core temperature is -7°C. -18-7 = freezer temperature is -18°C, and sample endpoint core temperature is -7°C. -29-7 = freezer temperature is -29°C, and sample endpoint core temperature is -7°C. -77-7 = freezer temperature is -77°C, and sample endpoint core temperature is -7°C. -12-12 = freezer temperature is -12°C, and sample endpoint core temperature is -12°C. -18-12 = freezer temperature is -18°C, and sample endpoint core temperature is -12°C. -29-12 = freezer temperature is -29°C, and sample endpoint core temperature is -12°C. -77-12 = freezer temperature is -77°C, and sample endpoint core temperature is -12°C. -18-18= freezer temperature is -18°C, and sample endpoint core temperature is -18°C. -29-18 = freezer temperature is -29°C, and sample endpoint core temperature is -18°C. -77-18 = freezer temperature is -77°C, and sample endpoint core temperature is -18°C. -29-29= freezer temperature is -29°C, and sample endpoint core temperature is -29°C. -77-29= freezer temperature is -77°C, and sample endpoint core temperature is -29°C. -77-77= freezer temperature is -77°C, and sample endpoint core temperature is -77°C. ^∗^^a-d^Treatments with different letters for freeze loss and thaw loss are significantly different (*P* ≤ 0.05). *n* = 9. Standarderrorforporenumbers = 9.09.

**Figure 7:**
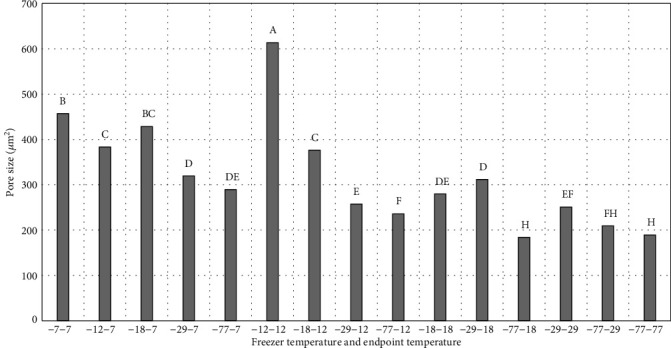
Pore size (*μ*m^2^) on peach slice surfaces. -7-7 = freezer temperature is -7°C, and sample endpoint core temperature is -7°C. -12-7 = freezer temperature is -12°C, and sample endpoint core temperature is -7°C. -18-7 = freezer temperature is -18°C, and sample endpoint core temperature is -7°C. -29-7 = freezer temperature is -29°C, and sample endpoint core temperature is -7°C. -77-7 = freezer temperature is -77°C, and sample endpoint core temperature is -7°C. -12-12 = freezer temperature is -12°C, and sample endpoint core temperature is -12°C. -18-12 = freezer temperature is -18°C, and sample endpoint core temperature is -12°C. -29-12 = freezer temperature is -29°C, and sample endpoint core temperature is -12°C. -77-12 = freezer temperature is -77°C, and sample endpoint core temperature is -12°C. -18-18 = freezer temperature is -18°C, and sample endpoint core temperature is -18°C. -29-18 = freezer temperature is -29°C, and sample endpoint core temperature is -18°C. -77-18 = freezer temperature is -77°C, and sample endpoint core temperature is -18°C. -29-29 = freezer temperature is -29°C, and sample endpoint core temperature is -29°C. -77-29 = freezer temperature is -77°C, and sample endpoint core temperature is -29°C. -77-77= freezer temperature is -77°C, and sample endpoint core temperature is -77°C. ^∗^^a-h^Treatments with different letters for freeze loss and thaw loss are significantly different (*P* ≤ 0.05). *n* = 9. Standarderrorforporesize = 16.73.

As previously discussed, ice crystal pore size and the number of ice crystals formed are directly impacted by freezing rate and reflected in drip loss. As expected, the surface ice crystal pore size was inversely related to freezing rate thus greater for peaches frozen at -7°C than -77°C while the pore numbers were directly related to freezing rate (Figures 6 and 7). And as just discussed, slower freezing caused large ice crystal to be formed in peaches, while fast freezing resulted in smaller ice crystal which leading to less damage to the peaches during freezing. Smaller ice crystals result in better frozen food quality while slow freezing creates large ice crystals particularly in extracellular space and causes tissue damage. Rapid freezing produces numerous small ice crystals that are more uniformly distributed both in intracellular and extracellular spaces [22, 23]. Rapid freezing is useful in fruit and vegetable products; however, temperature fluctuations during storage could cause recrystallization and loss of quality in frozen product [23].

**Figure 8:**
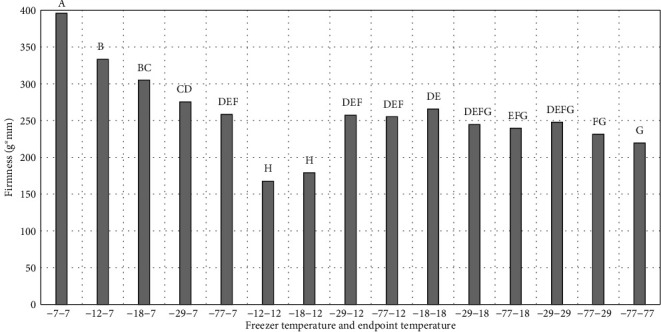
Razor blade shear (g∗mm) for fresh peach slices frozen at different rates and to different endpoint temperatures. -7-7 = freezer temperature is -7°C, and sample endpoint core temperature is -7°C. -12-7 = freezer temperature is -12°C, and sample endpoint core temperature is -7°C. -18-7 = freezer temperature is -18°C, and sample endpoint core temperature is -7°C. -29-7 = freezer temperature is -29°C, and sample endpoint core temperature is -7°C. -77-7 = freezer temperature is -77°C and sample endpoint core temperature is -7°C. -12-12 = Freezer temperature is -12°C, and sample endpoint core temperature is -12°C. -18-12 = freezer temperature is -18°C, and sample endpoint core temperature is -12°C. -29-12 = freezer temperature is -29°C, and sample endpoint core temperature is -12°C. -77-12 = freezer temperature is -77°C, and sample endpoint core temperature is -12°C. -18-18 = freezer temperature is -18°C, and sample endpoint core temperature is -18°C. -29-18 = freezer temperature is -29°C, and sample endpoint core temperature is -18°C. -77-18 = freezer temperature is -77°C, and sample endpoint core temperature is -18°C. -29-29 = freezer temperature is -29°C, and sample endpoint core temperature is -29°C. -77-29 = freezer temperature is -77°C, and sample endpoint core temperature is -29°C. -77-77= freezer temperature is -77°C, and sample endpoint core temperature is -77°C. ^∗^^a-d^Treatments with different letters for freeze loss and thaw loss are significantly different (*P* ≤ 0.05). *n* = 9. Standarderrorforshear = 10.99.

Peaches were generally lighter (higher *L*^∗^) and redder (higher *a*^∗^) in lower temperature freezers (faster freezing rates) compared to the higher freezer temperatures with slower freezing rate (Table 1). Variation in *b*^∗^ values resulted in a lack of significance for the freezing treatments. The chroma values ranged between 27.9 and 30.1, and hue ranged between 82.6 and 88.9 for all treatments with no freezing effect trends. Slower freezing resulted in peaches with higher shear force values compared to faster freezing rates (Figure 8). Lightness, redness, and shear force were directly related to freezer temperature/freezing rates reflecting the retention of water in the cellular structure of peaches. Darker appearance and increased firmness were likely due to formed larger ice crystals formed during slow freezing, which damaged the cellular reducing the turgor pressure and increasing firmness values of the samples.

Sensory panelists judged peaches similar to fresh peaches in appearance. But after a 24-hour thawing (at 3.33°C) cycle, all peaches exhibited browning despite prefreezing treatment with 2% of ascorbic acid. Freezing causes cell damage to the delicate organelles and membrane structures within the fruit. As one consequence, enzyme systems may be dislocated and released from organelles leading to enzyme activity with a variety of deteriorative reactions and effects, including browning. Moreover, water loss from the intracellular peach components impact turgidity increasing the intracellular solute concentration affecting pH and ionic strength on the unfrozen portion of the peach tissue. This concentration of solutes will negatively affect product quality [24, 25]. Browning of frozen peaches during thawing is caused by a combination of (1) damage of chloroplasts and chromoplasts by ice formation and volume expansion; (2) development of enzymatic browning; (3) changes in natural colorants, including the conversion of chlorophylls (green) to pheophytins (brown), complexation of anthocyanins and other pigments from leukoanthocyanins, and degradation of carotenoids [26, 27].

There was no difference between treatments for ascorbic acid antioxidant capacity (Table 2). The loss of ascorbic acid can be caused by long-term storage or high temperatures or physical damage to fruit by chill injury [28]. Sahari et al. [29] found no effect of freezing rate on vitamin C content of Iranian strawberries thus changes in antioxidant capacity in frozen foods is expected to be minimal.

### 3.2. Experiment 2. Long-Term Freezing Effects on Peach Quality

**Figure 9:**
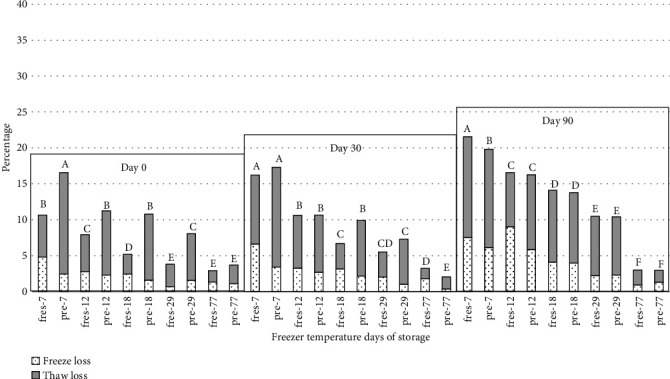
Weight loss during freezing and thawing and combined weight loss for fresh and prefrozen peach slices stored at different temperatures for 0, 30, and 90 days. *n* = 3. fres: fresh peaches; pre: prefrozen peaches. ^a-f^Values with different letters within each sampling day are significantly different (*P* ≥ 0.05). Standarderrorforcombinedfreezeandthawloss = 1.23.

**Figure 10:**
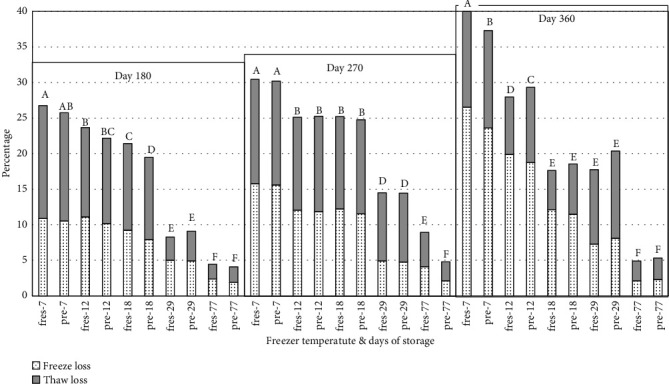
Weight loss during freezing and thawing and combined weight loss for fresh and prefrozen peach slices stored at different temperatures for 180, 270, and 360 days. *n* = 3. fres: fresh peaches; pre: prefrozen peaches. ^a-g^Values with different letters within each sampling day are significantly different (*P* ≥ 0.05). Standarderrorforcombinedfreezeandthawloss = 1.23.

Weight loss for peaches significantly increased for all storage temperatures over the 1-year storage study; however, the increase in weight loss was generally greater for peaches stored at higher temperatures compared peaches in lower temperatures (Figures 9 and 10). Except for day 0, the weight loss due to freezing and thawing did not differ between prefrozen and fresh peaches. Peaches frozen at 77°C had lower in weight loss than peaches frozen and held at all other temperatures on days 30, 90, 180, 270, and 360. Also, peaches frozen at -29°C had lower in weight loss than peaches held at 18°C, 12°C, and 7°C after 90, 180, 270, and 360 days of storage. There was no significant difference in weight loss for peaches held at -7°C and -12°C on days 90, 180, 270, and 360. Other researchers have also observed the weight loss of food after thawing [30–32]. Rao [30] found a direct relationship between weight loss and the amount of pectic substances, sugars, titratable acidity, ash, and minerals which found in the drip. Pukszta and Palich [31] indicated that weight loss in frozen strawberries increased with the length of and temperature variations during storage. Gormley [32] indicated that weight loss can be related to the loss of turgidity in fresh fruits due to the imbalance between the continuous loss of water by transpiration and lack of water uptake.

**Figure 11:**
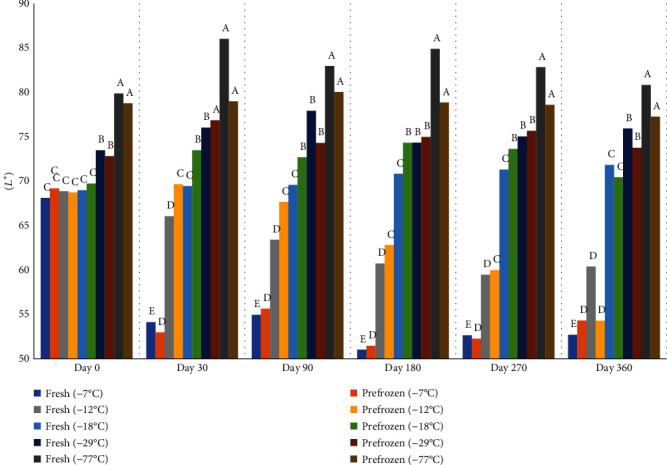
Average lightness (*L*^∗^) of fresh and prefrozen peaches held at different temperatures for 1 year. *n* = 9. ^a-e^Fresh samples within sampling day with different superscripts are significantly different (*P* < 0.05). ^a-e^Prefrozen samples within sampling day with different superscripts are significantly different (*P* < 0.05).

There was a direct relationship between lightness and lower freezing temperature; thus, the peaches frozen at lower temperatures became darker during storage (Figure 11). Peaches frozen at 7°C were darker than peaches frozen at all other temperatures at 30 days of storage. By 180 days of storage, peaches frozen at both 7°C and 12°C were darker than peaches frozen at the other temperatures. Furthermore, peaches held at -77°C were lighter than peaches in -29°C on days 30, 90, 180, 270, and 360, and peaches in -29°C were lighter than peaches held at 18°C, 12°C, and 7°C on days 30, 90, 180, 270, and 360. All samples enzymatically browned upon thawing after one week at freezer of -7°C; therefore, frozen peaches are best used for applications where they can be used in the frozen state and before thawing.

**Figure 12:**
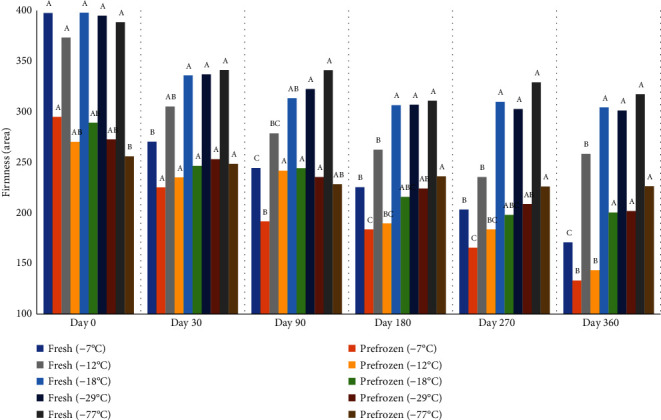
Average firmness (areag∗sec) of fresh and prefrozen peaches frozen at different temperatures and held for one year. ^a-e^Fresh samples within sampling day with different superscripts are significantly different (*P* < 0.05). *n* = 9. Standarderror = 16.9. ^a-e^Prefrozen samples within sampling day with different superscripts are significantly different (*P* < 0.05). *n* = 9. Standarderror = 13.9.

Firmness of peaches generally decreased during frozen storage regardless of the freezer temperature (Figure 12). Furthermore, fresh peaches were more firm than prefrozen peaches at all freezer temperatures. There was no difference in the firmness between peaches frozen at -77°C, -29°C, and -18°C on days 30, 90, 180, 270, and 360, and peaches in freezers at -29°C and -18°C were firmer than peaches held at -12°C and -7°C on days 180, 270, and 360. The firmness of peaches frozen at -7°C was lower than all other freezing temperatures. Higher freezer temperatures such as -12°C and -7°C will damage peaches' cell membranes caused by ice crystal volume expansion that reduces product integrity after thawing resulting in excessive drainage [33]. Lower freezing temperatures such as -77°C, -29°C, and -18°C can form a protective ice layer on the peach surface minimizing volume expansion during freezing [34]. Firmness is also related to the pectin content which has both water-soluble and insoluble fractions [35]. Therefore, loss of water-soluble pectin during thawing will reduce peach firmness [5].

**Figure 13:**
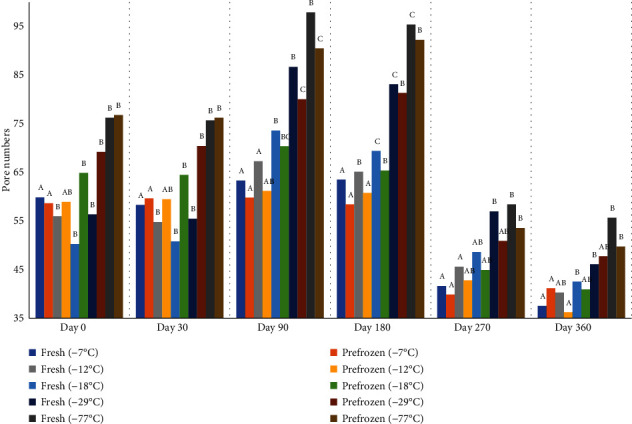
Scanning electron microscopy (pore numbers) of fresh and prefrozen peaches frozen and held for one year at different temperatures. *n* = 9. ^a-e^Fresh samples within sampling day with different superscripts are significantly different (*P* < 0.05). Standard error =5.7. ^a-e^Prefrozen samples within sampling day with different superscripts are significantly different (*P* < 0.05). Standarderror = 5.0.

**Figure 14:**
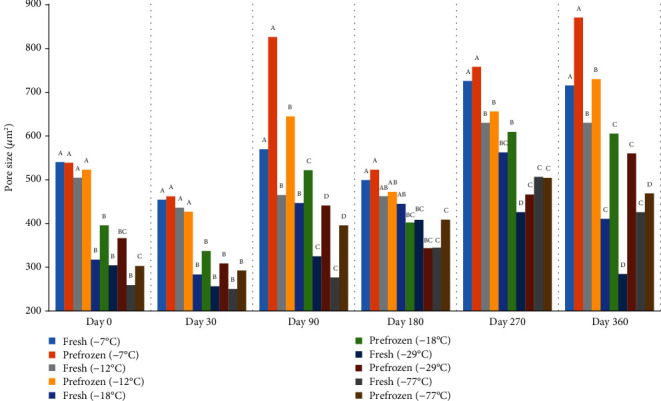
(pore size *μ*m^2^) 500 *μ*m of fresh and prefrozen peaches frozen and held for one year at different temperatures. *n* = 9. ^a-e^Fresh samples within sampling day with different superscripts are significantly different (*P* < 0.05). Standarderror = 28.1. ^a-e^Prefrozen samples within sampling day with different superscripts are significantly different (*P* < 0.05). Standarderror = 36.8.

**Figure 15:**
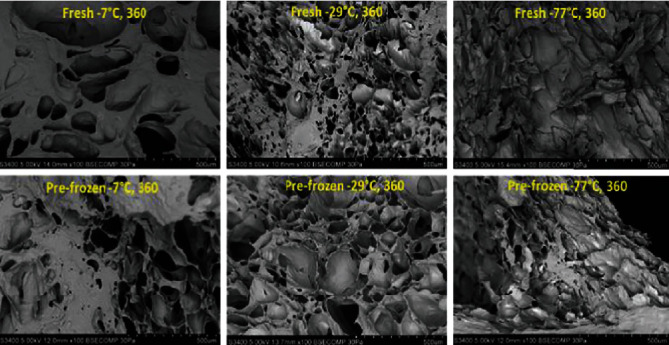
Scanning electron microscopy (pore size *μ*m^2^) (500 *μ*m) of fresh and prefrozen peaches surfaces. *n* = 9.

The number of pores decreased, and the size of pores increased during storage at all freezer temperatures (Figures 13 and 14, respectively). Changes in lightness and firmness reflect the changes in pore size and number. The larger pore size mirror decreases in firmness and lightness of peaches due to cell membrane damage. There was no difference in pore numbers for peaches held at -77°C and -29°C on days 30, 90, 180, 270, and 360. There was also no difference in peach pore numbers between freezers -29°C and -18°C on days 30, 90, 180, 270, and 360. The peaches frozen at lower temperatures of -77°C and -29°C had smaller pores than other freezers. But, the growth of ice crystal ruptures, pushes, and compresses the peaches cells. Micrographs of peach surfaces frozen from the fresh state and held from the commercially prefrozen state are visually smaller for the peach samples held at -77°C and -29°C compared to those held at -7°C (Figure 15). The nucleation of ice and the numbers and sizes of ice crystals formed depend on the freezing rate; slower rates of freezing will produce less nucleation, larger ice crystals, and more dislocation (migration) of water and ice; hence, the ice accumulates in large areas. Alternatively, faster rates will produce more nucleation, but smaller ice crystals [5].

**Figure 16:**
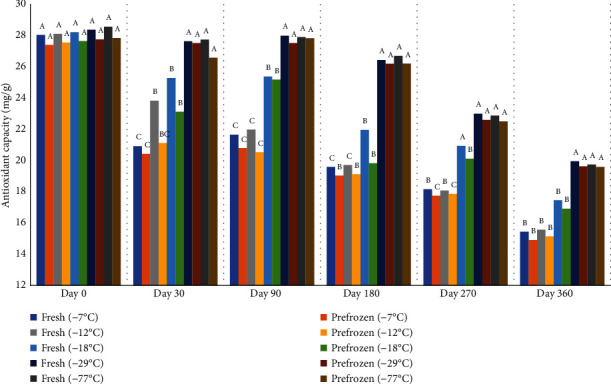
Ascorbic acid equivalent antioxidant capacity (mg/g) of fresh and prefrozen peaches frozen and held for one year at different temperatures. *n* = 9. ^a-e^Fresh samples within sampling day with different superscripts are significantly different (*P* < 0.05). Standarderror = 2.1. ^a-e^Prefrozen samples within sampling day with different superscripts are significantly different (*P* < 0.05). Standarderror = 1.8.

The antioxidant capacity of peaches significantly decreased over time (Figure 16). Peaches stored at -77°C and -29°C retained higher antioxidant capacity than other freezers in days 30, 90, 180, 270, and 360. There was no difference in peach antioxidant capacity held at -7°C and -12°C on days 90, 180, 270, and 360. However, not just ascorbic acid is responsible for the antioxidant properties of peaches but also phenolic content. The main bioactive compounds in fruits are vitamins A and C, carotenoids, and phenolics. These compounds give the foods their antioxidant properties [37]. De Ancos et al. [38] found that freezing process slightly affected total phenolic content in raspberry and a continuous decrease in the total ellagic acid content. The losses were attributed to the enzyme polyphenol oxidase released from the cell wall of the fruit during storage. The phenolic content in the peach pulp varies which is from 0.82 to 6.52 mg gallic acid equivalent [39]. The major loss of ascorbic acid observed at freezers -7°C and 12°C because of peach weight loss during thawing which decreased the level of ascorbic acid. Ascorbic acid is water soluble which could be affected by ice crystal formation. The degradation of ascorbic acid is affected during frozen storage by several factors such as pretreatments, type of food and packaging, freezing process, time, and temperature conditions [40]. Oxidation of ascorbic acid may be enzymatic or nonenzymatic. The enzyme mainly causing loss of vitamin C is ascorbate oxidase [37]. Many studies indicated that stability of ascorbic acid is higher in some fruits such as berries, citrus, and tomato. when the frozen storage temperature decreases [40, 41]. Sahari et al. [29] found that the loss of vitamin C was considerable at -12°C; however, vitamin C was higher at -18°C and -24°C [29].

**Figure 17:**
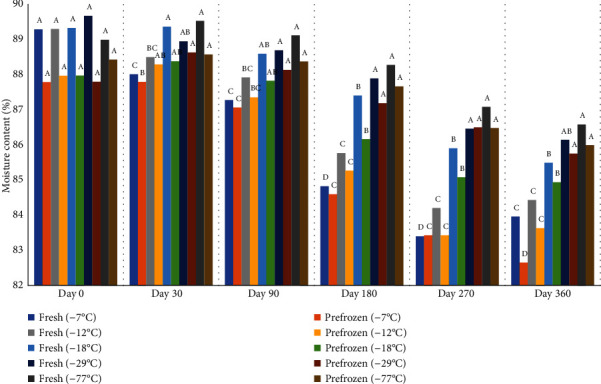
Average moisture content (%) of fresh and prefrozen peaches frozen and held for one year at different temperatures. *n* = 9.^a-e^Fresh samples within sampling day with different superscripts are significantly different (*P* < 0.05). Standarderrorforfreshpeaches = 4.5. ^a-e^Prefrozen samples within sampling day with different superscripts are significantly different (*P* < 0.05). Standarderrorforprefrozenpeaches = 3.6.

Moisture content of peaches decreased over time and was higher in peaches frozen and held at lower temperature (Figure 17). Lower moisture mirrored the loss of antioxidant capacity which may be due in part to the loss of water-soluble vitamins such as ascorbic acid. There was no difference in moisture content for peaches held at -77°C and -29°C on days 30, 90, 180, 270, and 360. Peaches in -77°C also had higher moisture content than peaches held in -18°C, -12°C, and -7°C freezers on days 180, 270, and 360.

**Figure 18:**
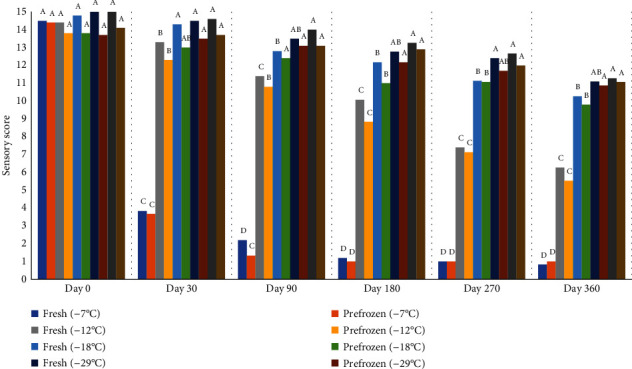
Sensory evaluation of fresh and prefrozen peaches frozen and held for one year at different temperatures. *n* = 9 (0 = extremely dislike to 15 = extremely like). *n* = 3. ^a-e^Fresh samples within sampling day with different superscripts are significantly different (*P* < 0.05). Standarderror = 0.4. ^a-d^Prefrozen samples within sampling day with different superscripts are significantly different (*P* < 0.05). Standarderror = 0.3.

Sensory scores for frozen peaches decreased during storage; however, peaches stored at -18°C, -29°C, and -77°C retained relatively high sensory scores through the one year storage cycle while peaches stored at -12°C and -7°C dropped below the “like” level (Figure 18). In fact, the peaches stored at -7°C dropped to 1 “extremely dislike” on the 1-15 scale. There was no significant difference in peaches' sensory scores when stored at -77°C and -29°C on days 30, 90, 180, 270, and 360. Peaches stored at -29°C were scored higher than those stored at -12°C and -7°C on days 30, 90, 180, 270, and 360. The quality attributes of peaches stored at -7°C were significantly lower than other freezers in days 30, 90, 180, 270, and 360.

## 4. Conclusions

Fast freezing at -29°C and -77°C generally maintained peach quality than slow freezing at other temperatures after freezing before thawing. However, all treatments displayed enzymatic browning after thawing. Once frozen, long-term frozen storage at -29°C and -77°C slowed the loss of nearly all quality attributes during the 12-month storage cycle compared to other storage temperatures. Interestingly, higher holding temperatures (-12°C and -18°C) were similar to lower temperatures (-29°C and -77°C) as far as sensory panel judgements through 90 days. Similar results were found for hexanal levels in that -12°C and -18°C showed minimal and similar hexanal levels as peaches stored at -29°C and -77°C through 90 days. Thus, peach quality can be maintained at these higher temperatures to potentially reduce energy costs while retaining quality during frozen storage.

## Figures and Tables

**Table 1:** Lightness (*L*^∗^) and redness (*a*^∗^) for fresh peaches frozen at different rates and to different endpoint temperatures.

Freezer temp	Endpoint core temperature									
	(-7°C)		(-12°C)		(-18°C)		(-29°C)		(-77°C)	
	*L* ^∗^	*a* ^∗^	*L* ^∗^	*a* ^∗^	*L* ^∗^	*a* ^∗^	*L* ^∗^	*a* ^∗^	*L* ^∗^	*a* ^∗^
-7°C	69.01^c^	1.64^i^								
-12°C	65.37^d^	1.95^gh^	68.07^c^	2.32^ef^						
-18°C	70.35^b^	1.79^hi^	67.51^c^	2.97^d^	67.91^c^	2.98^cd^				
-29°C	70.11^b^	2.01^cd^	69.61^bc^	3.15^bc^	70.12^b^	2.93^d^	72.57^b^	2.44^e^		
-77°C	65.03^d^	1.01^j^	69.42^bc^	2.04	70.54^b^	2.26^f^	72.41^b^	3.22^b^	79.5^a^	3.72^a^

^a-j^Values for each color parameter with different letters are significantly different (*P* ≤ 0.05). *n* = 9. Standarderrorfor*L* = 0.68 and for *a*^∗^ = 0.06.

**Table 2:** Ascorbic acid equivalent antioxidant capacity (mg/g peaches, wet basis) for thawed fresh peaches frozen at different temperatures.

Freezer temp	Endpoint core temperature				
	(-7°C)	(-12°C)	(-18°C)	(-29°C)	(-77°C)
-7°C	31.53 ± 1.89				
-12°C	32.64 ± 1.24	32.36 ± 2.61			
-18°C	32.53 ± 1.76	32.05 ± 2.24	32.09 ± 2.57		
-29°C	31.54 ± 2.09	32.33 ± 2.57	31.88 ± 2.49	32.43 ± 2.40	
-77°C	31.47 ± 2.33	31.19 ± 2.81	31.71 ± 2.33	31.39 ± 2.57	31.84 ± 2.89

Standarderrorforantioxidantcapacity = 0.79. *n* = 9.

## Data Availability

The data for this research is available upon request.
